# Step towards elimination of *Wuchereria bancrofti* in Southwest Tanzania 10 years after mass drug administration with Albendazole and Ivermectin

**DOI:** 10.1371/journal.pntd.0010044

**Published:** 2022-07-20

**Authors:** Jonathan Mnkai, Thomas F. Marandu, Jacklina Mhidze, Agatha Urio, Lucas Maganga, Antelmo Haule, Godfrey Kavishe, Elizabeth Ntapara, Nhamo Chiwerengo, Petra Clowes, Sacha Horn, Maureen Mosoba, Wilfred Lazarus, Abdallah Ngenya, Akili Kalinga, Alex Debrah, Friedrich Rieß, Elmar Saathoff, Christof Geldmacher, Achim Hoerauf, Michael Hoelscher, Mkunde Chachage, Inge Kroidl

**Affiliations:** 1 National Institute of Medical Research (NIMR)-Mbeya Medical Research Centre (MMRC), Mbeya, Tanzania; 2 University of Dar es Salaam -Mbeya College of Health and Allied Sciences (UDSM-MCHAS), Mbeya, Tanzania; 3 Division of Infectious Diseases and Tropical Medicine, University Hospital of the University of Munich (LMU), Munich, Germany; 4 National Institute of Medical Research (NIMR)–Headquarters, Dar es Salaam, Tanzania; 5 Kumasi Centre for Collaborative Research (KCCR) at the Kwame Nkrumah University of Science and Technology, Kumasi, Ghana; 6 German Center for Infection Research (DZIF), partner site Munich, Munich, Germany; 7 German Center for Infection Research (DZIF), partner site Bonn-Cologne, Bonn, Germany; 8 Institute of Medical Microbiology, Immunology and Parasitology, University Hospital Bonn, Bonn, Germany; 9 German-West African Centre for Global Health and Pandemic Prevention (G-WAC), Partner Site Bonn, Bonn, Germany; Federal University of Agriculture Abeokuta, NIGERIA

## Abstract

**Background:**

Lymphatic filariasis is a mosquito transmitted parasitic infection in tropical regions. Annual mass treatment with ivermectin and albendazole is used for transmission control of *Wuchereria bancrofti*, the infective agent of lymphatic filariasis in many African countries, including Tanzania.

**Methodology:**

In a general population study in Southwest Tanzania, individuals were tested for circulating filarial antigen, an indicator of *W*. *bancrofti* adult worm burden in 2009 before mass drug administration commenced in that area. Seven annual rounds with ivermectin and albendazole were given between 2009 and 2015 with a population coverage of over 70%. Participants of the previous study took part in a follow-up activity in 2019 to measure the effect of this governmental activity.

**Findings:**

One thousand two hundred and ninety nine inhabitants of Kyela district in Southwest Tanzania aged 14 to 65 years who had participated in the study activities in 2009 were revisited in 2010/11 and 2019. Among this group, the prevalence of lymphatic filariasis of the 14–65 years olds in 2009 was 35.1%. A follow-up evaluation in 2010/11 had shown a reduction to 27.7%. In 2019, after 7 years of annual treatment and an additional three years of surveillance, the prevalence had dropped to 1.7%, demonstrating successful treatment by the national control programme. Risk factors for *W*. *bancrofti*-infection were the occupation as farmer, male sex, and older age. Most infected individuals in the 2019 follow-up study already had a positive test for filarial antigen in 2009 and/or 2010/11.

**Conclusions:**

This data supports the findings of the Tanzanian Neglected Tropical Disease Control Programme (NTDCP), who conducted Transmission Assessment Surveys and found an impressive reduction in the prevalence of LF in children. Our results complement this data by showing a similar decrease in prevalence of LF in the adult population in the same area. The elimination of LF seems achievable in the near future.

## Author summary

Lymphatic Filariasis (LF) is a helminth infection which can lead to disfiguring lymphedema, recurrent pain and inflammation, chronic disability and social stigma. Hence, LF is among neglected tropical diseases targeted for elimination as a public health problem. In 2000, the WHO launched its Global Programme to Eliminate Lymphatic Filariasis (GPELF). Large scale annual treatment of all eligible individuals in regions or countries where the infection is present was implemented to reduce the transmission of LF. In an area with a high prevalence of lymphatic filariasis in Southwest Tanzania, annual treatment with albendazole and ivermectin was conducted between 2009 and 2015. The effect of the government treatment was verified by our group in a carefully selected cohort of 1299 individuals who had participated in study activities both before and after mass drug administration. A significant drop in the prevalence from 35.1% to 1.7% was demonstrated by measuring the circulating filarial antigen by ELISA. The majority of the remaining infected individuals showed a reduction of the *W*. *bancrofti* worm burden. This government treatment was verified as an enormous step towards the elimination of lymphatic filariasis in Tanzania.

## Introduction

Despite 20 years of WHO’s global efforts to eliminate lymphatic filariasis (LF), this disfiguring disease still remains a global health burden [1–5]. *Wuchereria bancrofti* is one of the three helminth species that causes LF and is responsible for 90% of LF infections. Before larger treatment programmes started, LF was present in most of the 21 regions of Tanzania with up to 63.8% of individuals testing positive for *W*. *bancrofti* circulating filarial antigen (CFA), a marker for filarial infection [6]

For the past two decades, the Global Alliance to Eliminate Lymphatic Filariasis (GPELF) has been using annual mass drug administration (MDA) as a strategy to control and ultimately eliminate the transmission of LF [7–9]. Since the launch of GPELF, the number of LF infected individuals has dropped from ~ 199 million in 2000 to 51 million in 2018 [10]. In Africa, there has been a reduction from 74 million individuals to 10 million who are currently LF infected [10,11]. Several LF endemic regions have reported a significant reduction in LF prevalence; 17 of 72 filariasis endemic countries have successfully achieved the elimination of LF as a public health problem and three of them (Egypt, Togo and Malawi) are within Africa [10,12]. In October 2009, the Tanzanian National Lymphatic Filariasis Elimination Programme (NLEFP) started MDA treatment programs in the Mbeya region in Southwest Tanzania with the annual distribution of albendazole (400mg) and ivermectin (150–200μg/kg). At that time, a *W*. *bancrofti* prevalence of 32% was reported by the Tanzanian NTD programme for Kyela district in Mbeya region among individuals above 15 years of age. We have previously published the moderate prevalence reduction after two years of treatment in Kyela district as measured by CFA [13]. However, since treatment with ivermectin and albendazole mainly kill the microfilariae but only mildly damage adult worms with a life-span of 5–7 years, effectiveness of treatment can only be evaluated after a longer treatment period [14,15]. For example, a longitudinal study from northern Tanzania showed only moderate reductions of CFA positivity in people aged one year and above, after two annual drug distributions (from 53.3% to 51.4%), but a significant drop to 44.9% and 19.6% after four and six years of treatment, respectively [16,17]. Therefore, our current study aimed to further assess the prevalence of *W*. *bancrofti* in the region 10 years after LF treatment had begun.

## Methods

### Ethics statement

The RHINO study was approved by the Mbeya Medical Research Ethics Committee (GB.152/377/01/194), the Tanzanian National Health Research Ethics Committee (NIMR/HQ/R.8a/Vol. IX/2856) and the Ethics Committee of the medical faculty of the University of Munich (project ID: 18–377). Prior to enrolment, each RHINO participant was fully briefed on the study and provided written informed consent regarding study participation. Parents consented for their children below 18 years of age and, in addition, children who were able also signed their own assent forms.

### Study population and study design

The study was a follow-up of the population based EMINI (**E**valuation and **M**onitoring of the **I**mpact of **N**ew **I**nterventions, http://www.mmrp.org/projects/cohort-studies/emini.html) cohort study, which was conducted at the National Institute for Medical Research (NIMR)—Mbeya Medical Research Centre (MMRC) ([13,18–22]. The study was carried out in selected villages from nine geographically distinct areas in the Mbeya region (S1 Fig) which included 10% of the households of each selected village. In one of the study sites (Kyela), testing for adult *W*. *bancrofti* was done from archived samples collected annually between 2007 and 2011 using the TropBio ELISA as the microfilariae data were not available. During the ongoing RHINO study (**R**isk of **H**IV **I**nfections through **N**ematode **O**rganism), new data was collected in the Kyela district between March and August 2019 from 1299 previous EMINI participants aged 14–65 years. Characteristics of RHINO study participants are highlighted in Table 1.

**Table 1:** Comparison of EMINI participants who were followed up in the RHINO study with those who were lost to follow-up.

			Lost to follow-up	Followed up in RHINO	p-value*
**# of subjects**					
		n	856	1299	
**Sex**					0.6315
	female	n (%)	440 (51.4)	654 (50.3)	
	male	n (%)	416 (48.6)	645 (49.7)	
	missing	n	0	0	
**Age in 2009 in years**					
		median (IQR)	17.0 (10.5 to 26.9)	17.5 (10.1 to 30.9)	0.2829
	missing	n	0	0	
**SES rank**					
		median (IQR)	3.3 (1.3 to 6.7)	3.1 (1.0 to 6.1)	0.0010
	missing	n	49	22	
**HIV status**					0.0047
	neg.	n (%)	746 (87.3)	1182 (91.1)	
	pos.	n (%)	109 (12.7)	116 (8.9)	
	missing	n	1	1	
**LF status**					0.7792
	neg.	n (%)	632 (73.8)	952 (73.3)	
	pos.	n (%)	224 (26.2)	347 (26.7)	
	missing	n	0	0	

* p-value for differences between RHINO participants and those lost to follow-up.

** Social economic status (SES). Wilcoxon rank sum test for continuous variables (Age and SES) and chi-squared test for binary variables (all others)

### Sample collection

Blood, urine and stool samples from participants in the Kyela site of the EMINI study had been collected annually from 2007 until 2011, as previously described [13]. Between March and August 2019, previous EMINI participants were revisited in order to evaluate treatment impact. Bio-banked samples from March 2009 from the 1299 EMINI subjects who participated the RHINO follow-up study were used to estimate the prevalence of LF in the same cohort directly before the government anti-LF treatment program commenced in Kyela in October 2009. For the 2019 follow-up visit, 10 ml of blood was collected from each study participant, during morning hours in EDTA tubes (BD Vacutainer) and immediately stored at room temperature (18–20°C). Plasma and whole blood cells were separated within 24 hours and subsequently stored at -80°C and -20°C, respectively. All tests were performed at the NIMR-MMRC laboratories in Mbeya, Tanzania.

### Filarial Antigen testing

The CFA was detected by using the commercially available ELISA kit TropBio Og4C3 (Cellabs, New South Wales, Australia), using 100 μl of the collected plasma as performed in the previous study [13]. Briefly, 100μl of plasma was tested for the presence of CFA by measuring optical density (OD) values which were then compared to the OD from the standardized amounts of filarial antigen also supplied with the kit. The results of these measurements were shared with Cellabs and analysed according to their suggestions, documented in an updated version of the manufacturer´s instruction (version LF2.3) which states that:*”For ELISA plates with standard 2 OD not within range of +/- 10% of 0*.*35*, *a default cut-off OD of 0*.*35 shall be used*.*”* As all ELISA showed a standard 2 above the proposed level a cutoff of OD 0.35 was used throughout. Positive values were defined as low if the OD was between 0.35 and 0.8, medium if the OD was 0.8 to 1.0 and high positive for OD greater then1.0. Values were measured by the Sunrise ELISA instrument (Tecan).

### Statistics

Statistical analyses were performed using Stata statistics software (version 15.1; Stata Corp., College Station, TX). Pearson´s chi-squared test was used to compare binominal outcomes between groups and to compare CFA positivity before and after treatment in all participants. The Wilcoxon rank-sum test was used to compare CFA positivity before and after treatment in those individuals who participated in all surveys. Furthermore we performed uni- and multi-variable log link binomial regression analyses with robust variance estimates in order to assess potential risk factors for LF infection.

## Results

### Characteristics of the study population

All households in Kyela who previously participated in the EMINI study ([13]) were contacted to enrol inhabitants aged 14 to 65 years into the RHINO study. Of 2187 potentially eligible individuals with previous data for LF, 1299 (60%) were enrolled into the follow-up activity and 856 (40%) were not found or chose not to participate.

Reasons for not participating were primarily due to a permanent move of the household. About 2.5% of eligible participants refused a follow-up. A comparison of the data from the EMINI study shows that age, sex and LF prevalence of individuals who participated in the follow-up activity were similar to those who did not (Table 1). Of note, the LF-prevalence is age-dependent and the study participants were ~ 10 years younger during the EMINI survey.

Table 2 details the demographic characteristics of the RHINO study participants. Of the 1299 participants, 654 (50.3%) were females, the median age was 27 years, 12 individuals (0.9%) suffered from lymphedema of the leg and 12 (1.9%) of the men had a visible scrotum hydrocele. The prevalence of HIV was 13.5% and was twice as high in women (17.9%) than in men (9.1%, p = <0.0001).

**Table 2:** Characteristics of the study population in 2019 (RHINO study).

			all	female	male
# of subjects					
		n	1299	654	645
**Age in years**					
	14 to <25 yrs	n (%)	530 (40.8)	247 (37.8)	283 (43.9)
	25 to <45 yrs	n (%)	535 (41.2)	281 (43.0)	254 (39.4)
	45 to < = 66 yrs	n (%)	234 (18.0)	126 (19.3)	108 (16.7)
	missing	n	0	0	0
**HIV status**					
	neg.	n (%)	1123 (86.5)	537 (82.1)	586 (90.9)
	pos.	n (%)	176 (13.5)	117 (17.9)	59 (9.1)
	missing	n	0	0	0
**LF status**					
	neg.	n (%)	1277 (98.3)	649 (99.2)	628 (97.4)
	pos.	n (%)	22 (1.7)	5 (0.8)	17 (2.6)
	missing	n	0	0	0
**Lymphedema**					
	absent	n (%)	1287 (99.1)	647 (98.9)	640 (99.2)
	present	n (%)	12 (0.9)	7 (1.1)	5 (0.8)
	missing	n	0	0	0
**Hydrocele**					
	absent	n (%)	626 (98.1)	N/A	626 (98.1)
	present	n (%)	12 (1.9)	N/A	12 (1.9)
	missing	n	7	N/A	7

### Prevalence of LF infection 10 years after the start of mass anti filarial drugs administration

We determined the prevalence reduction of LF (measured by the level of CFA) after seven rounds of MDA (2009 until 2015) and an additional three years in which transmission assessment surveillance took place. In 2009, before the first round of MDA started in the region the prevalence of LF among all participants of the initial study who were aged 14–65 years at this time was 35.1% (392 of 1116). In 2011, two years post-MDA introduction, the LF prevalence in the same age group had dropped to 27.7% (196 of 707). In a second analysis, only the subgroup of individuals who participated in the activities in 2009, 2011 and 2019 were considered and a prevalence of 33.7% (231 of 686) for 2009 and 29.3% (122 of 417) for 2010/11 was calculated. In 2019, 1299 of previous EMINI subjects who participated in the RHINO study were screened for the presence of CFA as a marker of an active filarial infection. The overall LF prevalence among these participants was 1.7% (22/1299) which was significantly lower than the measurement pre- and two years post-introduction of MDA (p<0.0001).

**Fig 1:**
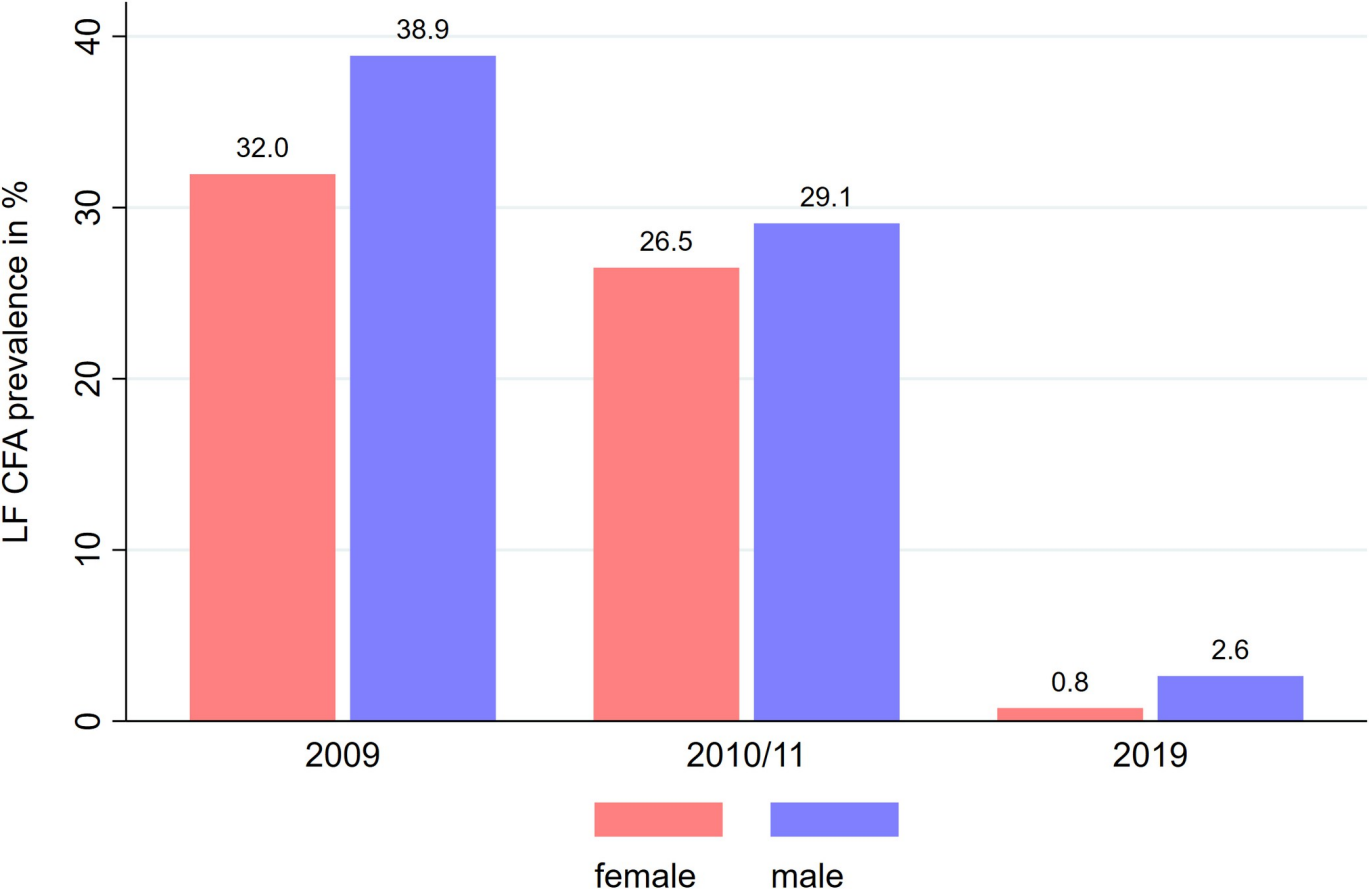
LF prevalence categorized by sex. The LF prevalence defined by the level of circulating filarial antigens was measured by Trop Bio ELISA in the two EMINI surveys (2009 and 2010/11) and the RHINO study (2019) in individuals 14 to 65 years in Kyela district. LF prevalence is higher in male compared with female participants in all three surveys.

When the prevalence was compared by sex, LF was less prevalent in female than male participants in the 2009 EMINI survey (32.0% vs 38.9%, p = 0.016), in the 2010/11 follow-up (26.5% vs 29.1%, p = 0.442) and the RHINO study (0.8% vs 2.6%, p = 0.008, Fig 1).

**Fig 2:**
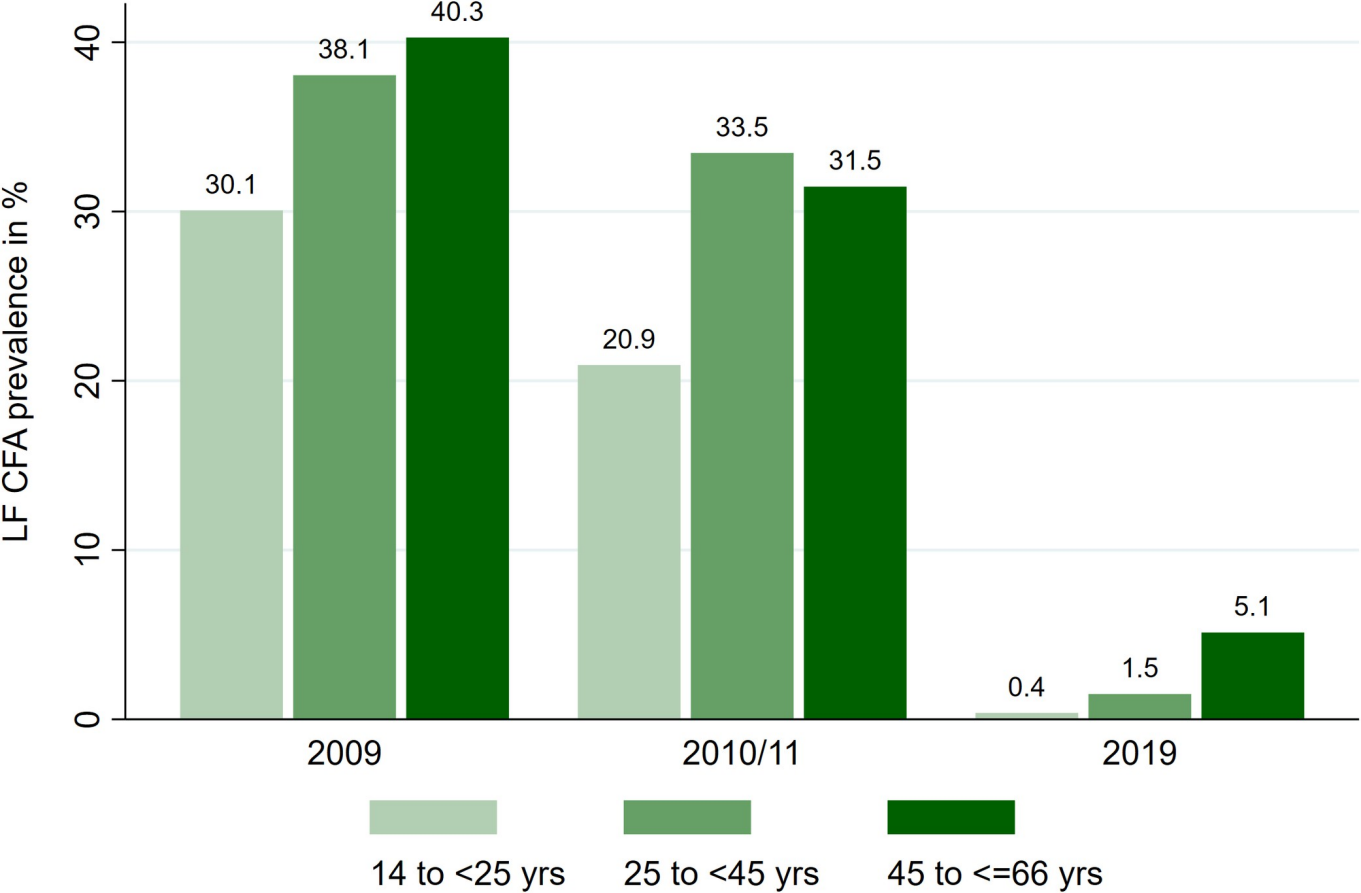
LF prevalence categorized by age group. The LF prevalence (defined by the level of circulating filarial antigens) is increasing with older age in the two EMINI surveys (2009 and 2010/11) as well as in the RHINO study (2019).

Stratification by age showed that in the 2009 and 2011 EMINI surveys, LF prevalence was lower in young individuals within the age of 14 to <25 years. Similarly, during the RHINO survey in 2019, LF prevalence was the lowest within the same group (14 to <25 years) while the 45 to <65 years group had the highest prevalence (Fig 2).

Multi-variable analysis confirmed that *W*. *bancrofti* infection was associated with increasing age, male sex and filarial infection during the previous study (Table 3). Five of the 22 infected individuals in 2019 were women and four of them worked as farmers. Of the 17 infected men, 15 were farmers (S2 Fig).

**Table 3:** Association of various factors with LF result in 2019 Uni- and multi-variable log-link regression results adjusted for within-household clustering.

				univariable			multivariable		
Covariate	N	N-pos.	%-pos.	RR	95% CI	p-value	RR	95% CI	p-value
**Sex**									
**female** *	654	5	0.76	1.00	- -	-	1.00	- -	-
**male**	645	17	2.64	3.45	(1.27 to 9.33)	0.0148	3.45	(1.29 to 9.20)	0.0133
**Age in 2019**									
**14 to <25 yrs** *	530	2	0.38	1.00	- -	-	1.00	- -	-
**25 to <45 yrs**	535	8	1.50	3.96	(0.84 to 18.67)	0.0817	1.96	(0.42 to 9.02)	0.3894
**45 to < = 66 yrs**	234	12	5.13	13.59	(3.03 to 60.95)	0.0007	6.94	(1.71 to 28.16)	0.0067
**Previously LF positive 2007 to 2011**									
**neg.** *	952	5	0.52	1.00	- -	-	1.00	- -	-
**pos.**	347	17	4.90	9.33	(3.48 to 24.98)	<0.0001	6.16	(2.49 to 15.26)	<0.0001

N = number of observations; N-pos. = number of positives; %-pos. = percent positive; RR = risk ratio; 95% CI = 95% confidence interval.

* reference stratum

### Adult worm burden

**Fig 3:**
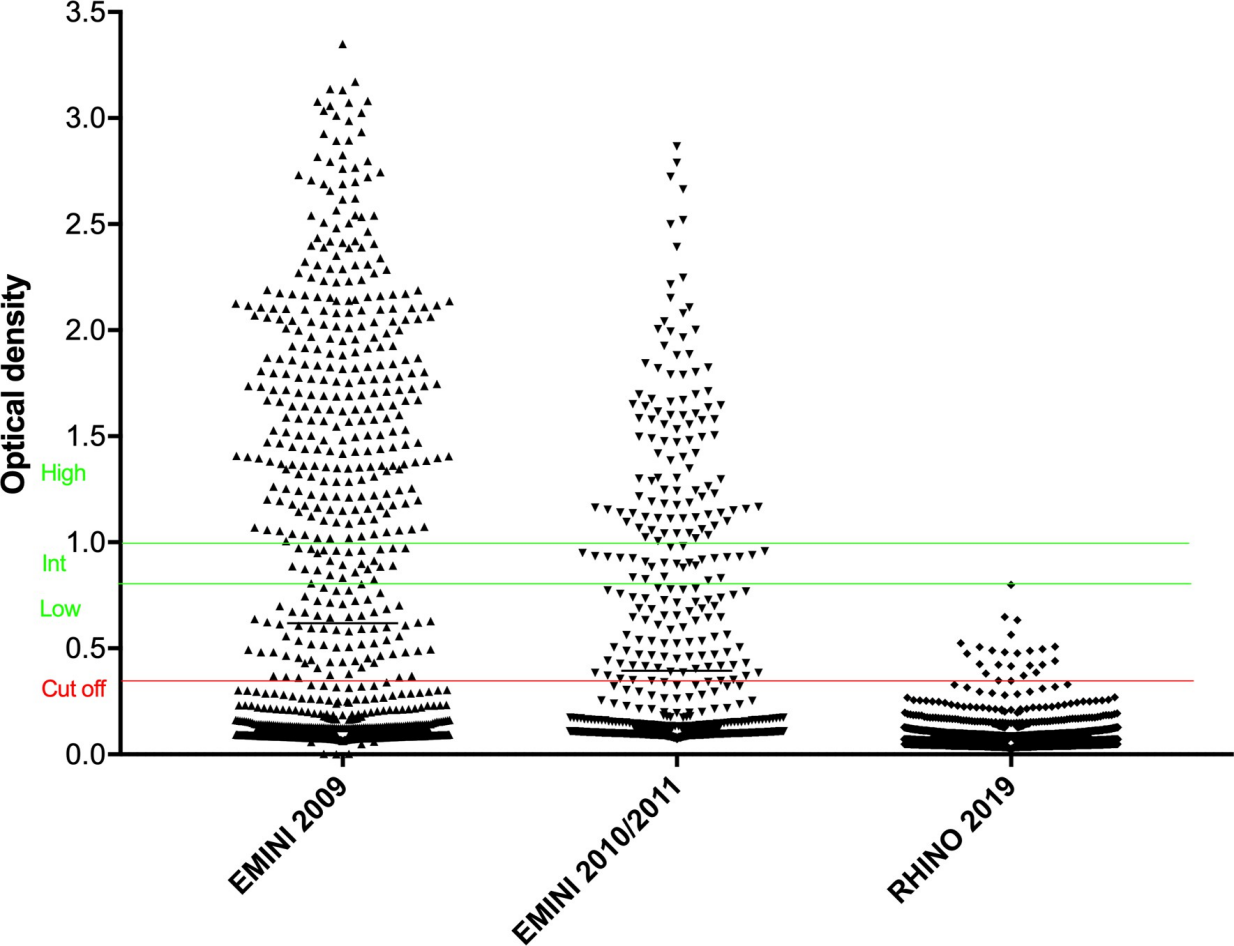
OD values of the TropBio ELISA of plasma samples from individuals 14 to 65 years of age are shown. The TropBio ELISA is measuring the circulating filarial antigen of the adult filarial worm. Values above OD 0.35 are considered as positive, with low positive results between OD 0.35 and 0.8, intermediate positive results between OD 0.8 and 1.0, and high positive results above the OD of 1.0. Most filarial infected individuals in 2009 showed high positive results, reflecting a high adult worm burden. In 2019 infected individuals had low positive ODs indicating a low number of adult worms.

The CFA level was analysed using the OD values generated from the ELISA assay of plasma samples from 1299 RHINO participants from 2009, 2010/11 and 2019 (Fig 3).

The antigen level was graded according to the manufacturer’s instructions, with ODs between above the cutoff (OD 0.35) and OD 0.8 being low positive, a result between OD 0.8 and OD 1.0 medium positive, an OD above 1.0 high positive indicating high levels of antigen.

In 2009, the prevalence of LF among the EMINI/RHINO participants who were then aged 14 to 65 years was 35.1% (392 of 1116). Of the 392 positive samples, 76% were high positive, 8% medium positive and 16% low positive. In 2010/11 27.7% (196 of 707) of the participants were CFA positive, of them 52.5% had an OD > 1.0 indicative of high antigen levels, 12.3% medium positive, 35% low positive results. The follow-up in 2019 revealed no high or medium positive values, but rather all 22 CFA positive individuals had low positive results (Fig 3).

**Fig 4:**
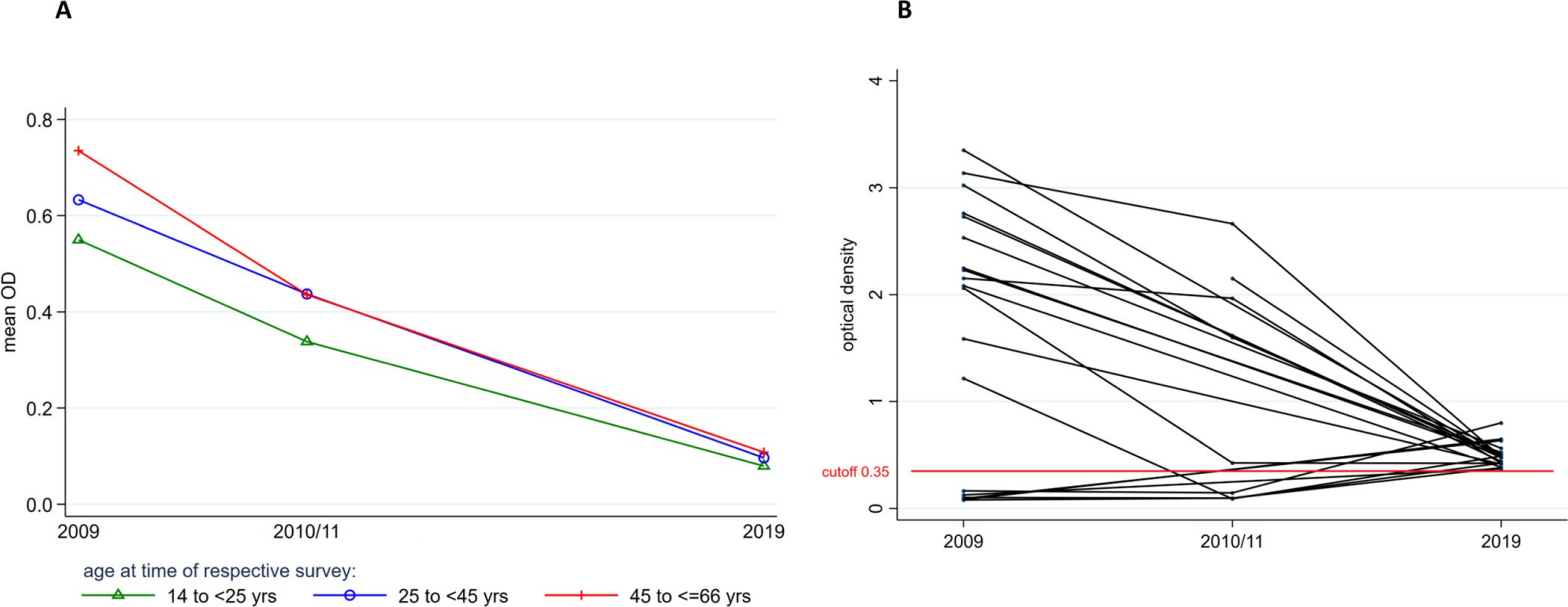
(A) Mean OD values over time categorized by age group.The OD values declined over time in all age groups. (B) OD values of Trop Bio ELISA from 2009, 2010/11 and 2019 from individuals who tested positive in 2019. Most of the remaining filarial infected individuals in 2019 had a positive result of the TropBio ELISA during the surveys in 2009 or 2010/11. Despite being still positive a significant drop of the OD was noted. Only four individuals had been tested negative before, but showed positive results in 2019.

Overall, the mean OD values by age group declined from 0.55, 0.63, 0.74 to 0.34, 0.44, 0.44 (38%, 30%, 40.5%) between 2009 and 2010/11, then to 0.08, 0.10, 0.11 (85.4%, 84.1%, 85.1%) in 2019 in participants within the age group range of 14 to <25, 25 to <45 and 45 to < = 66 years respectively (Fig 4A). The mean OD of CFA positive individuals was 1.55 (SD 0.70) in 2009, 1.11 (SD 0.56), in 2010/11 and 0.49 (SD 0.10) in 2019. Of the 22 individuals with positive CFA results in 2019, 18 had high antigen levels during the EMINI 2009 and 2010/11 surveys and there were 4 new infections of previously uninfected participants (Fig 4B).

### Incidence of Lymphatic Filariasis

The annual LF incidence rate was 1.45 per 100 person-years among the group of EMINI-RHINO participants between 2008 and 2009, before MDA activities commenced. From 2011 to 2019, four new infections occurred among 947 previously uninfected individuals, leading to an incidence of 0.05 per 100 person years, showing a 27-fold reduction in new infections.

## Discussion

Since the global program to eliminate lymphatic filariasis was launched in 2000, MDA programmes with anti-filarial drugs have globally reduced the number of infected individuals [10,23]. However, it is important to measure and control the sustainability of the treatment success after the campaign has ended. The WHO has established tools like the transmission assessment surveys (TAS), which are conducted by the countries some years after the treatment completion [24]. However, as TAS is focusing on measuring the CFA in six to seven year old school children, reports from independent institutions as well as evaluations among the adult population are valuable additions.

It is estimated that adult filarial worms live for 5 to 7 years and therefore MDA programmes usually treat areas over five or more years [14]. As ivermectin and albendazole do not impact adult worms, the prevalence reduction is achieved predominantly by interfering with microfilarial transmission which prevents new infections. In Mbeya district, the national MDA programme commenced in late 2009 and distributed anti-filarial drugs annually until 2015. We previously published data from Kyela region that showed a moderate reduction of the LF prevalence after the first two years of the anti-filarial MDA programme from 35.1% in 2009, to 27.7% in 2011 among the individuals aged 14 to 65 years [13].

In the study presented here, the study participants were again visited in 2019, after seven years of annual treatment and additional three years of surveillance. Our results show a dramatic reduction in LF prevalence from 35.1% in 2009, to 1.7% in 2019 among the individuals aged 14 to 65 years. The intriguing feature in this cohort is that the very same people have been visited in 2009, 2010/11 and 2019, thus excluding a sampling bias of this study. Admittedly this approach adds an age bias instead, but since older age is associated with higher LF-prevalence, this age bias should lead to an increased prevalence, while our study shows a greatly reduced prevalence. We have also presented analytical data which, in addition, shows a significant reduction in the sera CFA levels of RHINO study participants (Figs 3 and 4), indicating a reduced adult worm burden in the infected individuals.

Matching the OD values of the TropBio ELISA from the previous and the current study we found that 18 of the 22 remaining infected individuals (81.8%) were still CFA seropositive, but at the same time showed a significantly reduced antigen load. This shows that the treatment has an effect even among the infected individuals (Figs 3 and 4). It can be concluded that these individuals were reached by the MDA programme but that the treatment efficacy was not optimal or treatment incomplete. This is a benefit of using a quantitative ELISA instead of a qualitative measurement. Only four of 22 (18%) of the infected individuals in 2019 were new cases with low positive values for the TropBio ELISA. Major risk factors for the infection with *W*. *bancrofti* were male sex, older age, and occupation as a farmer.

Other publications have described the effect of MDA programmes which use albendazole and ivermectin in Tanzania. A limited effect on the prevalence of LF measured by CFA was seen after two rounds of treatment in a study from northern Tanzania [16]. However, the adult worm burden of the infected individuals dropped significantly, which was shown by lower OD levels of the samples measured by TropBio ELISA. In the same area, a re-evaluation took place after four, six and eight years of treatment and a significant reduction in LF prevalence was demonstrated [17,25], similar to our results presented here. In a recent assessment of that area in northern Tanzania, a CFA prevalence of 5.8% was measured, using the Filarial Test Strip, a qualitative method to test for filarial antigen [26]. The same study also showed a higher LF prevalence in males than females and a prevalence increase with age, similar to our findings. Within our study population, we observed the lowest CFA prevalence in the youngest group of participants in the age range of 14 to <25. In addition, males had a significantly higher LF prevalence than females pre- and 10 years post- MDA (Fig 1).

Our data are highly consistent with the reports from the Tanzanian Neglected Tropical Disease Control Programme (NTDCP). The WHO recommends a pre-Transmission Assessment Survey (pre-TAS) for all people aged 5 years and above, and three additional TAS at set intervals among 1^st^ and 2^nd^ grade school children aged 6–7 years. Ending the MDA programmes is recommended when TAS shows a prevalence of filarial antigen below 2%. After passing the TAS-1 in 2015 with 0.36% prevalence (6 of 1635), MDA was stopped in Kyela district (personal communication with NTDCP). Of note, our reported LF prevalence in Kyela is higher than that reported by NTDCP, most likely because our study participants were between 14 and 65 years of age while the NTDCP survey only included children aged six to seven years. Moreover, we observed four new LF infections in the recent survey, indicating a possible resurgence, long after MDA has stopped in the study area. In our study a negative correlation with age was found: the prevalence of LF was 5.1% among 45 to 65 year old, 1.5% among the 25 to 45 year old and 0.4% among the 14 to 25 year old participants. The study area of Kyela district is bordering Malawi and the Tanzanian programme to eliminate LF would not have been successful if Malawi had not joined the global elimination activity. The bordering Karonga district of Northern Malawi had reported an LF prevalence of 25% among adults in 2002 [27]. After several years of treatment Malawi had eliminated LF in 2020 [11].

## Conclusion

Ten years after an MDA programme to treat *W*. *bancrofti* infections was implemented in Kyela district, we observed a vast reduction in LF prevalence in the area. Most of the few remaining infected individuals had been positive for CFA before the MDA programme was conducted, and only a few new infections occurred. Major risk factors for infection with *W*. *bancrofti* were male sex, older age, and being a farmer. The elimination of Lymphatic Filariasis in Kyela district seems achievable in the near future.

## Supporting information

S1 Fig Study area. The study was conducted in the south-western part of Tanzania (Fig S1 red rectangle). A general population study was performed in nine study areas (black polygons). Data for this study were collected in Kyela (red circle) situated close to Lake Nyassa (Image from Elmar Saathoff as previously published in [20,21]). The continent-level and country-level shape-files used Vector Map Level 0 (VMap0) data, which can be downloaded at https://mdl.library.utoronto.ca/collections/geospatial-data/vector-map-level-0-vmap0. Elevation data were retrieved from NASAs Shuttle Radar Topography Mission (SRTM) version 2.1, which can be found at https://www2.jpl.nasa.gov/srtm/. Both datasets are in the public domain.
(TIF)

S2 Fig Profiling Farmers among individuals with LF. Farmers (dark grey bars), non-farmers (light grey bars)
(TIFF)
