# Supplementary figures and images for: Step towards elimination of Wuchereria bancrofti in Southwest Tanzania 10 years after mass drug administration with Albendazole and Ivermectin
Source: PLoS Negl Trop Dis. 2022 Jul 20;16(7):e0010044. doi: 10.1371/journal.pntd.0010044 (PMC9342735; doi:10.1371/journal.pntd.0010044)

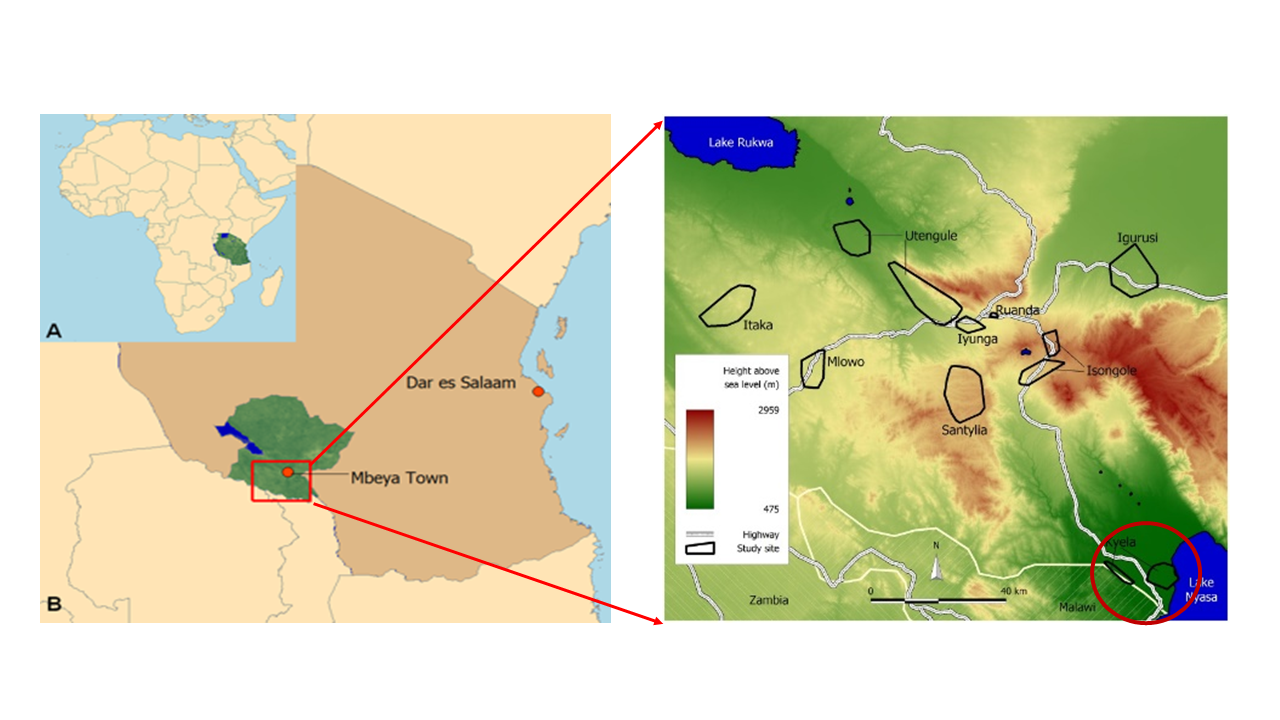

Supplement: S1 Fig — The study was conducted in the south-western part of Tanzania (Fig S1 red rectangle). A general population study was performed in nine study areas (black polygons). Data for this study were collected in Kyela (red circle) situated close to Lake Nyassa (Image from Elmar Saathoff as previously published in [20,21]). The continent-level and country-level shape-files used Vector Map Level 0 (VMap0) data, which can be downloaded at https://mdl.library.utoronto.ca/collections/geospatial-data/vector-map-level-0-vmap0. Elevation data were retrieved from NASAs Shuttle Radar Topography Mission (SRTM) version 2.1, which can be found at https://www2.jpl.nasa.gov/srtm/. Both datasets are in the public domain. (TIF) [file pntd.0010044.s001.tif]

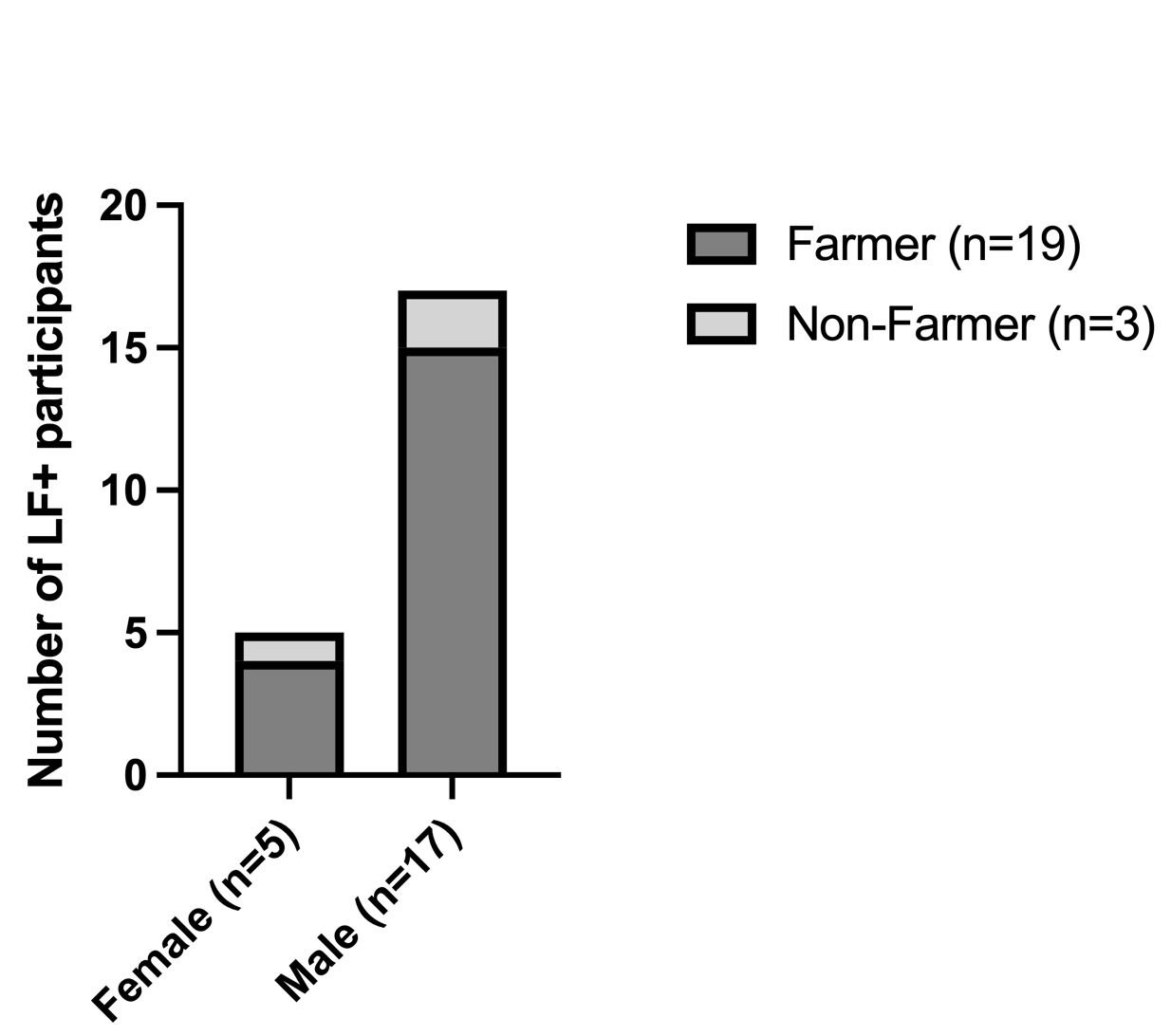

Supplement: S2 Fig — Farmers (dark grey bars), non-farmers (light grey bars) (TIFF) [file pntd.0010044.s002.tiff]
